# Motile and non-motile *Listeria* species adopt distinct ecological and evolutionary strategies to achieve broad geographic ranges across soil ecosystems

**DOI:** 10.1093/ismejo/wrag158

**Published:** 2026-06-19

**Authors:** Ying-Xian Goh, Shannon Hepp, Kevin J Cummings, Martin Wiedmann, Jingqiu Liao

**Affiliations:** Department of Civil and Environmental Engineering, Virginia Tech, Blacksburg, VA 24061, United States; Center for Emerging, Zoonotic, and Arthropod-Borne Pathogens, Virginia Tech, Blacksburg, VA 24061, United States; Global Change Center, Virginia Tech, Blacksburg, VA 24061, United States; Department of Civil and Environmental Engineering, Virginia Tech, Blacksburg, VA 24061, United States; Department of Public and Ecosystem Health, Cornell University, Ithaca, NY 14853, United States; Department of Food Science, Cornell University, Ithaca, NY 14853, United States; Department of Civil and Environmental Engineering, Virginia Tech, Blacksburg, VA 24061, United States; Center for Emerging, Zoonotic, and Arthropod-Borne Pathogens, Virginia Tech, Blacksburg, VA 24061, United States; Global Change Center, Virginia Tech, Blacksburg, VA 24061, United States

**Keywords:** *Listeria*, geographic ranges, motility, selection, dispersal

## Abstract

Broad geographic ranges often reflect ecological versatility and are associated with lower extinction risk. Motility is a key physiological and ecological trait in bacteria. However, how some motile and non-motile bacteria achieve broad geographic ranges remains poorly understood. Here, we analyzed the genomes of 141 *Listeria welshimeri* and 90 *Listeria booriae* isolates systematically obtained from soils, representing widespread motile and non-motile species, respectively. We show that *L. welshimeri* lacks clear phylogeographic structure, suggesting minimal geographic barriers to dispersal. Its wide distribution is likely associated with enhanced motility and effective host colonization that facilitate wildlife-driven dispersal, particularly by regional-terrestrial birds. This pattern is supported by positive selection on flagellar and chemotaxis genes, strong associations with wildlife movement patterns, and close genomic relatedness between soil and wild bird isolates. In contrast, *L. booriae* displays clade endemism and a strong distance–decay relationship, suggesting dispersal limitation. Despite lacking a dispersal advantage, *L. booriae*’s wide distribution appears to be linked to genomic flexibility and metabolic versatility that support adaptation to diverse environmental conditions, especially those shaped by iron concentration and precipitation. This is evidenced by its large, open pangenome characterized by abundant and diverse metabolic pathways and broad substrates utilization capacity; pronounced positive selection on genes involved in inorganic ion, amino acid, and coenzyme transport and metabolism; and strong associations between gene richness and abiotic factors as well as bacterial community composition. These findings suggest distinct genomic foundations and ecological and evolutionary mechanisms underlying the success of motile and non-motile cosmopolitan bacteria in soil ecosystems.

## Introduction

Geographic range, the spatial extent over which a species occurs, is a key determinant of microbial ecology, associating with population connectivity, resilience, and ecosystem functioning [1, 2]. Species with broader ranges typically exploit a wider variety of habitats or resources, reflecting greater niche breadth, and are often considered generalists [3–5]. Although geographic range expansion can entail fitness costs, such as reduced performance in the original habitat due to niche-expanding mutations [6] or slower adaptation to novel conditions [7], generalists commonly gain a competitive advantage under environmental fluctuations, making them less prone to extinction when habitats are disturbed [8, 9]. Evolutionary processes such as pangenome expansion [10] and recombination [11] have been proposed to facilitate bacterial survival across diverse environments; however, large-scale field data that mechanistically link genomic features and ecological and evolutionary processes to their widespread geographic distributions remain limited.

Free-living bacteria can be broadly categorized into motile and non-motile forms. Motility confers multiple ecological advantages, including microhabitat exploration, efficient nutrient acquisition, toxin avoidance, and habitat and host colonization [12–14]. Experimental studies show that motile bacteria expand at rates determined by growth and habitat size [15], with chemotaxis enabling directed range expansion [16]. However, these benefits are counterbalanced by substantial energetic costs, including the biosynthesis and maintenance of flagellar structures and adenosine triphosphate (ATP) expenditure for locomotion [12]. Trade-offs between chemotactic benefits and energetic burden may constrain the net advantage of motility, particularly in complex or turbulent environments [17], leaving open the question of whether mechanistic advantages observed under laboratory conditions translate into broad-scale geographic distribution in natural habitats. In contrast, non-motile bacteria lack active movement and may have a reduced capacity for dispersal, host colonization, and rapid relocation to favorable microhabitats. This suggests that widespread non-motile taxa may evolve alternative strategies to achieve broad geographic ranges.

*Listeria* is a bacterial genus commonly present across diverse environments [18, 19]. By systematically screening 1004 soil samples collected across the contiguous United States, our previous study characterized soil-dwelling *Listeria* phylogroups along an endemic-to-cosmopolitan gradient and detected pronounced environmental selection in shaping genomic flexibility in cosmopolitan phylogroups [20]. However, how the mechanisms underlying the broad distributions of cosmopolitan *Listeria* species with and without motility differ remain unknown.

Here, we compared *Listeria welshimeri*, a species that is motile via peritrichous flagella [21], and *Listeria booriae*, a non-motile species [22], both of which displayed comparably broad geographic range sizes in soil across the United States [20]. Through an in-depth genomic and ecological analysis of 141 *L. welshimeri* and 90 *L. booriae* isolates [20], we identified evidence of distinct genomic foundations and ecological and evolutionary strategies that these two species adopt to achieve success in soils. *L. welshimeri* may rely on movement and the colonization of wildlife hosts, particularly regional-terrestrial birds, to facilitate long-distance dispersal. In contrast, *L. booriae*, which exhibited strong dispersal constraints, appears to offset the absence of motility by maintaining an extremely open pangenome enriched in signatures related to metabolism that enables adaptation to diverse environmental conditions, especially those shaped by iron (Fe) concentration and precipitation. Together, these findings advance our mechanistic understanding of microbial biogeography by distinguishing the genomic foundations, ecological processes, including wildlife-mediated dispersal and environmental filtering, and evolutionary processes, including positive selection, that underlie broad geographic ranges in motile and non-motile species.

## Materials and methods

### Genomic data of *Listeria welshimeri* and *Listeria booriae* isolates, environmental data, and bacterial community data

Existing genome assemblies of 141 *L. welshimeri* and 90 *L. booriae* isolates that we previously obtained from minimally disturbed soil in natural environments across the United States [20] were subjected to secondary analysis in this study. The genome assemblies of these two species were previously analyzed along with other species in the context of *Listeria* pangenome evolution but have not been used to directly compare between *L. welshimeri* and *L. booriae*. Soil sampling followed a systematic approach that achieved a relatively even spatial coverage across the United States [20]. Methods for bacterial isolation, DNA extraction, whole genome sequencing (WGS), WGS data preprocessing, genome assembly, gene prediction, orthologous gene identification, and functional annotation [i.e. assignment of functional categories for orthologous genes based on Clusters of Orthologous Groups (COGs) and Kyoto Encyclopedia of Genes and Genomes (KEGG)] for *L. welshimeri* and *L. booriae* soil isolates were previously described [20]. *L. welshimeri* and *L. booriae* co-occurred in 18 samples, and each species was detected across multiple samples (*L. welshimeri, n* = 71; *L. booriae, n* = 49). Isolates of the same species detected within the same sample represent different strains with distinct *sigB* allelic types [20]. Each isolate was therefore treated as an independent observation in downstream analyses.

Additionally, WGS was performed for nine *L. welshimeri* isolates recovered from wild regional-terrestrial birds, including rose-breasted grosbeak and rock pigeon, admitted to the Janet L. Swanson Wildlife Hospital at Cornell University, New York [23]. Following our data deposition, these isolates remained the only *L. welshimeri* wildlife isolates with WGS data available in the National Center for Biotechnology Information (NCBI) database. Genomic DNA was extracted using the NEBExpress T4 Lysozyme and Monarch Spin gDNA Extraction Kit (New England Biolabs) following the manufacturer’s protocol. DNA quality and concentration were assessed using a NanoDrop spectrophotometer and Qubit fluorometer, respectively. All samples met standard quality thresholds (A260/280 ≈ 1.80; A260/230 ≈ 2.0) and were sequenced on an Illumina MiSeq platform (2 × 250 bp paired-end reads) at the Duke University Center for Genomic and Computational Biology. WGS data preprocessing, genome assembly, gene prediction, and gene family identification followed the workflow previously described [20] with minor modifications to ensure comparability (see Supplementary Information for details). Quality statistics for wild bird isolates are summarized in Supplementary Table S1.

The environmental data comprised 34 variables (also referred to as “abiotic factors”): three geolocation variables (latitude, longitude, elevation), 17 soil physicochemical variables [moisture, total nitrogen (TN), total carbon (TC), pH, organic matter (OM), concentrations of aluminum (Al), calcium (Ca), copper (Cu), Fe, potassium (K), magnesium (Mg), manganese (Mn), molybdenum (Mo), sodium (Na), phosphorous (P), sulfur (S), and zinc (Zn)], four climate variables [precipitation, wind speed, maximum (Max.), and minimum (Min.) temperatures (temp.)], and 10 land use variables [proportional coverage of open water, barren land, forest, shrubland, grassland, cropland, pasture, wetland, and developed (Dev) open spaces with either >20% or <20% impervious (IMP) surfaces in the surrounding landscape]. Descriptions of environmental data acquisition and processing were previously described [20].

Soil bacterial community composition (also referred to as “biotic factors”) was characterized by 16S rRNA gene amplicon sequencing. Methods for DNA extraction, 16S ribosomal RNA (rRNA) gene amplicon sequencing, sequencing data preprocessing, operational taxonomic unit (OTU) identification, taxonomic classification, calculations of Shannon index, Pielou’s evenness index, and weighted UniFrac distances were reported in our previous study comparing *Listeria* populations from natural and food-associated environments [24]. Both abiotic and biotic factors were subjected to secondary analysis in the present study to assess their differences between samples positive for *L. welshimeri* and *L. booriae* as well as their associations with gene content.

### Phylogenetic trees, genetic similarities, and geographic distribution

To investigate phylogeographic structure, phylogenetic trees based on core single nucleotide polymorphisms (SNPs) were constructed for three groups: (i) *L. welshimeri* soil isolates, (ii) *L. booriae* soil isolates, and (iii) *L. welshimeri* soil and wild bird isolates. Core SNPs were identified using kSNP4 [25], and maximum likelihood trees were generated using RAxML v8.2.12 [26] under the GTR + G + I substitution model with ascertainment bias correction and 1000 bootstrap replicates. Trees were visualized using iTOL v7 [27]. To assess genomic similarity, pairwise average nucleotide identity (ANI) was computed using pyani v0.3.0-alpha in Python v3.6.8 for these three groups. In addition, Jaccard similarity indices were calculated using the gene presence/absence matrices for groups (i) and (ii).

Geographic distributions of *L. welshimeri* and *L. booriae* soil isolates, grouped by major clades (i.e. monophyletic groups that share a recent common ancestor) on the phylogenetic tree, were visualized using a Mercator projection with the Basemap Matplotlib Toolkit v1.2.1 in Python v3.6.8. Pairwise geographic distances between isolates were calculated from GPS coordinates using the geopy module in Python v3.6.8. Mean geographic distances within each major clade were then computed and compared between the two species using a two-sided Mann–Whitney (MW) *U* test.

### Pangenome characterization and statistical comparison of genomic features

Core genes are defined as those found in all genomes of a given species, whereas accessory genes are defined as those present in only a subset of genomes [10]. The pangenome refers to the complete repertoire of genes present across all genomes within each species, encompassing both core and accessory genes [10]. To estimate the pangenome size for soil-derived *L. welshimeri* and *L. booriae* based on an assumed 100 genomes each, we applied the power law function *cN^γ^* previously calculated [20], which models the relationship between the number of genomes (*N*) and total orthologous genes (i.e. pangenome size). The specific functions used were *n*_pan_ = 2572 *N*^0.133^ for *L. welshimeri* and *n*_pan_ = 2937 *N*^0.208^ for *L. booriae* [20].

To characterize genomic features, we analyzed genome size, gene richness (i.e. total number of unique ortholog genes per genome), nucleotide diversity (π), and KEGG pathway abundance and diversity for each species. Species-level π was calculated using popGenStat [28] on gene alignments generated with MUSCLE v3.8.31 [29]. Annotated genes were further classified into four major KEGG pathways [i.e. (1) metabolism, (2) genetic information processing, (3) environmental information processing, and (4) cellular processes] and into their corresponding subgroups (*n* = 22; 12, 4, 2, and 4 subgroups for major pathways 1–4, respectively; Supplementary Table S2). The Shannon index was calculated for all pathways combined (referred to as “overall biological pathway”) and within each major pathway and subgroup, based on their relative abundance. All genomic features were compared between *L. welshimeri* and *L. booriae* soil isolates using two-sided MW *U* tests. For the comparison of KEGG subgroup pathway abundance and diversity, Benjamini–Hochberg false discovery rate (BH-FDR) correction was applied to account for multiple testing. The overlap and unique KEGG Orthology (KO) annotations between *L. welshimeri* and *L. booriae* were assessed separately for the core and accessory genomes. In addition, KO annotations from the core genomes of *L. welshimeri* and *L. booriae* were mapped and visualized using iPath3.0 [30].

To assess the genomic similarities between monophyletic *L. welshimeri* soil and wild bird isolates, Kruskal–Wallis (KW) tests followed by pairwise two-sided MW *U* tests were used to compare ANI among (i) within-soil, (ii) soil-wild bird, and (iii) within-wild bird isolates. Genome size, gene richness, and guanine-cytosine (GC) content were also compared between soil and wild bird isolates using two-sided MW *U* tests.

### Positive selection detection and functional enrichment analysis

Soil isolates of *L. welshimeri* and *L. booriae* were subjected to positive selection detection using the methods as previously described [20] (see Supplementary Information for details). Only genes with an adjusted *P* < .05 in the positive selection analysis and no detectable recombination were considered to be undergoing positive selection. Functional enrichment analysis was performed using a binomial distribution model as previously described [24] to identify COGs significantly enriched among core and accessory genes under positive selection for each species.

### Metabolic modeling and phenotypic simulations

Genome-scale metabolic models were reconstructed from protein sequences predicted by Prodigal v2.6.3 [31] using CarveMe v1.6.6 [32] with the carve workflow and Gurobi optimizer v12.0.3 [33] under default parameters. To characterize the metabolic capacity of each isolate, simulations were performed in Python v3.12.12 using the COnstraints-Based Reconstruction and Analysis (COBRApy) library v0.30.0 [34]. To identify essential nutrients (i.e. external nutrients required for growth due to a lack of *de novo* biosynthetic capacity), mixed-integer linear programming was first employed to determine the absolute mathematical minimum number of nutrients required for a threshold growth rate of 0.01 h^−1^ [32]. These candidates were subsequently validated by testing for growth failure when each nutrient was individually omitted from a complete simulated medium. To identify utilizable substrates (i.e. substrates that support biomass production beyond the essential requirements), a universal master list of potential substrates was constructed by aggregating all exchange reactions present across all reconstructed models. For each isolate, we defined a minimal essential nutrient set required for a basal growth rate of 0.1 h^−1^ [32], followed by a systematic supplementation screen. A substrate was classified as utilizable if its individual addition to this baseline medium enabled a growth flux exceeding 0.01 h^−1^ [32]. Differences in the total number of utilizable substrates and the proportions of individual utilizable substrates between species were evaluated using two-sided MW *U* and Fisher’s exact tests with BH-FDR correction, respectively. Utilizable substrates showing significant differences (adjusted *P* < .05) were annotated based on their corresponding Biochemical, Genetic, and Genomic (BiGG) knowledgebase exchange IDs and subsequently categorized according to their functions.

### Environmental characteristics and distance–decay relationships

Multidimensional scaling (MDS) analyses based on Euclidean distances for abiotic factors and weighted UniFrac distances for OTUs followed by permutational multivariate analysis of variance (PERMANOVA), two-sided MW *U* tests for individual abiotic factors and co-occurring bacterial taxa followed by BH-FDR correction, a two-sided MW *U* test for Shannon and Simpson’s indices of bacterial community were employed to compare environmental conditions (abiotic and biotic) between soil samples positive for *L. welshimeri* and *L. booriae*.

To identify the influence of abiotic and biotic factors on the gene richness of soil-derived *L. welshimeri* and *L. booriae*, variation partitioning analysis (VPA) and Spearman’s rank correlation analyses were conducted. Two VPA models were constructed: (a) one that included three groups of abiotic variables—soil properties, climate, and surrounding land use—and (b) another that included two groups of variables, abiotic factors and biotic factors represented by relative abundances of bacterial phyla. To enhance model performance, we evaluated multi-collinearity among predictor variables using variance inflation factors (VIF). Variables with the highest VIF were iteratively removed until all remaining predictors had VIF <10 [35]. This procedure retained 22 and 42 variables for VPA models (a) and (b), respectively (Supplementary Table S3), to quantify the proportion of variance in gene richness explained by each group of selected variables within each species. Adjusted *R*^2^ values were computed using the vegan package 2.6-4 in R and visualized in a Venn diagram. Spearman’s rank correlations followed by BH-FDR correction were applied to assess associations between gene richness, both overall and for each COG, and each of the 34 abiotic factors, 25 bacterial phyla, 176 genera, and 170 species.

To infer dispersal limitation, distance–decay relationships were examined by regressing geographic distance and two measures of genomic similarity: ANI and Jaccard similarity. A linear regression was interpreted as evidence of a distance–decay relationship when it met all three criteria: an *R*^2^ > 0.2, a negative slope, and *P* < .05.

### Ecological connectivity modeling

To evaluate the potential for wildlife-mediated dispersal of *L. welshimeri* and *L. booriae*, four common wildlife vectors of *Listeria*: large mammals (e.g. deer, boar), small mammals (e.g. rat, mouse), regional-terrestrial birds (e.g. pigeon, crow), and continental-aquatic birds (e.g. goose, gull; Supplementary Table S4) were selected to model ecological connectivity, defined as the degree to which the landscape facilitates or impedes the movement of specific vectors or ecological processes [36, 37]. Initial landscape resistance classifications for each wildlife model were performed at a 30 m resolution using the 2024 National Land Cover Database (NLCD) data [38] with the details provided in the Supplementary Information. To enable continental-scale analysis and assess the effect of spatial grain on resistance distance calculation, these layers were aggregated to a 5, 3, and 1 km spatial resolutions using an average resampling approach. A 1 km resolution was ultimately selected to maximize the detection of localized landscape barriers and environmental features. Pairwise functional connectivity (resistance distance) was estimated using circuit theory [39, 40]. Genomic distance was calculated as 1 – ANI. Linear regression was used to quantify the proportion of genomic variation explained *R*^2^ by resistance distance for *L. welshimeri* and *L. booriae* across all ecological connectivity models.

## Results

### Soil-derived *Listeria booriae* exhibits phylogeographic structure and greater genetic diversity than *Listeria welshimeri*

**Figure 1:**
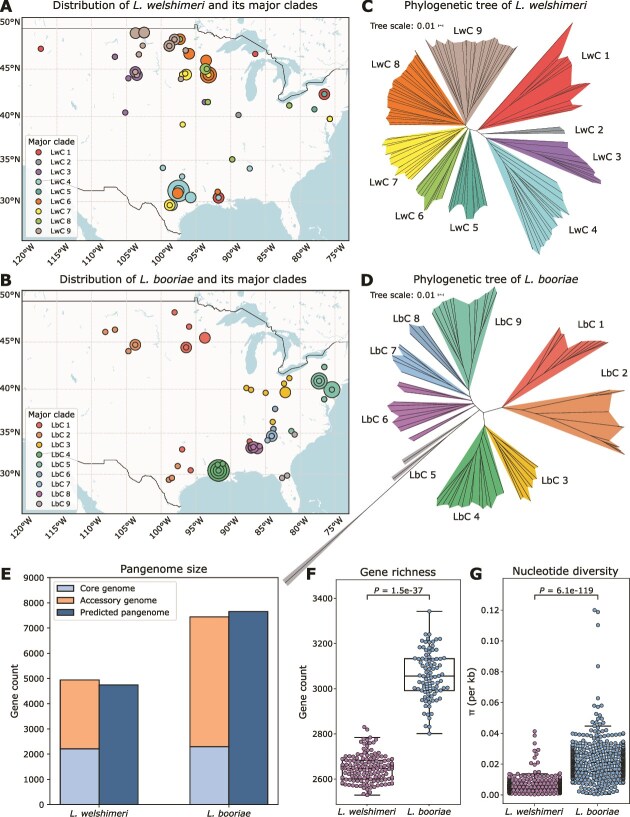
Phylogeographic structure and genomic features of soil-dwelling *L. welshimeri* and *L. booriae*; (A and B) geographic distribution of (A) *L. welshimeri* and (B) *L. booriae* isolates in soil across the contiguous United States, color-coded by major clades identified in c and d; circle sizes indicate the number of isolates per clade at each site; (C and D) unrooted maximum likelihood phylogenetic trees of (C) 141 *L. welshimeri* soil isolates and (D) 90 *L. booriae* soil isolates based on core SNPs; branches are color-coded by major clades, with “Lw” and “Lb” indicating *L. welshimeri* and *L. booriae*, respectively; (E) pangenome sizes of *L. welshimeri* and *L. booriae*, divided into core and accessory genomes, with predicted pangenome sizes estimated from 100 genomes per species using the power law function *cN^γ^* described previously [20]; (F and G) (F) gene richness and (G) nucleotide diversity (π) compared between *L. welshimeri* and *L. booriae*; box plots display the interquartile range (IQR) with the median indicated as a line and whiskers extending to 1.5 times the IQR; two-sided MW *U P*-values are annotated.

Our previous work showed that *L. welshimeri* and *L. booriae* were the most widespread motile and non-motile *Listeria* phylogroups detected in systematically collected, minimally disturbed natural soils across the contiguous United States [20]. Among 1004 soil samples, 89 (8.9%) and 67 (6.7%) were positive for *L. welshimeri* and *L. booriae*, respectively [20]. Although *Listeria monocytogenes* is also motile and exhibited a slightly higher overall prevalence (11.8%) than *L. welshimeri*, our previous systematic phylogenomic analysis revealed that *L. monocytogenes* comprises three species-level phylogroups (lineages I, II, and III) which substantially differ in genomic and ecological traits [20, 41]. At the phylogroup level, *L. welshimeri* was more prevalent than the most common *L. monocytogenes* lineage III (7.7%) and was therefore selected to represent the most widespread motile *Listeria* species in this study. Both *L. welshimeri* and *L. booriae* exhibited comparable broad geographic distributions (853 and 850 km, respectively; Fig. 1A and B). Core-SNP phylogenies revealed that *L. welshimeri* can be classified into nine major clades (Fig. 1C), whose members were geographically intermixed (Fig. 1A). *L. booriae* also comprised nine major clades (Fig. 1D) but exhibited clear geographic structuring. Its major clades were strongly confined to particular regions, showing evidence of clade-level endemism (Fig. 1B). To further examine this pattern, pairwise geographic distances between isolates within each major clade were calculated for each species. The mean geographic distances within major clades ranged from 234 to 963 km and 33 to 730 km for *L. welshimeri* and *L. booriae*, respectively (Supplementary Figure S1A) and were significantly larger in *L. welshimeri* (MW *U P* = .022; Supplementary Figure S1B). These findings indicate that although both *L. welshimeri* and *L. booriae* are cosmopolitan species with a comparably widespread distribution, they show distinct geographic and population structures.

To assess whether the observed population structures corresponded to differences in genomic content between the two species, we characterized their pangenomes. A total of 4941 orthologous genes in *L. welshimeri* (2209 core and 2732 accessory genes) and 7443 in *L. booriae* (2298 core and 5145 accessory genes) were identified (Fig. 1E). To avoid the potential bias caused by unequal sample size, we predicted the pangenome size given 100 genomes for each species based on the pangenome curves, where a more open pangenome was observed in *L. booriae* (Supplementary Figure S2A). The predicted pangenome size was 4745 for *L. welshimeri* and 7654 for *L. booriae.* Consistent with this result, *L. booriae* had significantly higher gene richness (MW *U P* = 1.5e-37; Fig. 1F) and larger genome size (*P* = 1.5e-37; Supplementary Figure S2B). Furthermore, it had significantly greater nucleotide diversity (*P* = 6.1e-199; Fig. 1G). Together, these results show that *L. booriae* has greater genomic diversity and flexibility than *L. welshimeri*.

### Distinct pathway enrichment and positive selection signatures highlight metabolic versatility in *Listeria booriae* and cell motility adaptation in *Listeria welshimeri*

**Figure 2:**
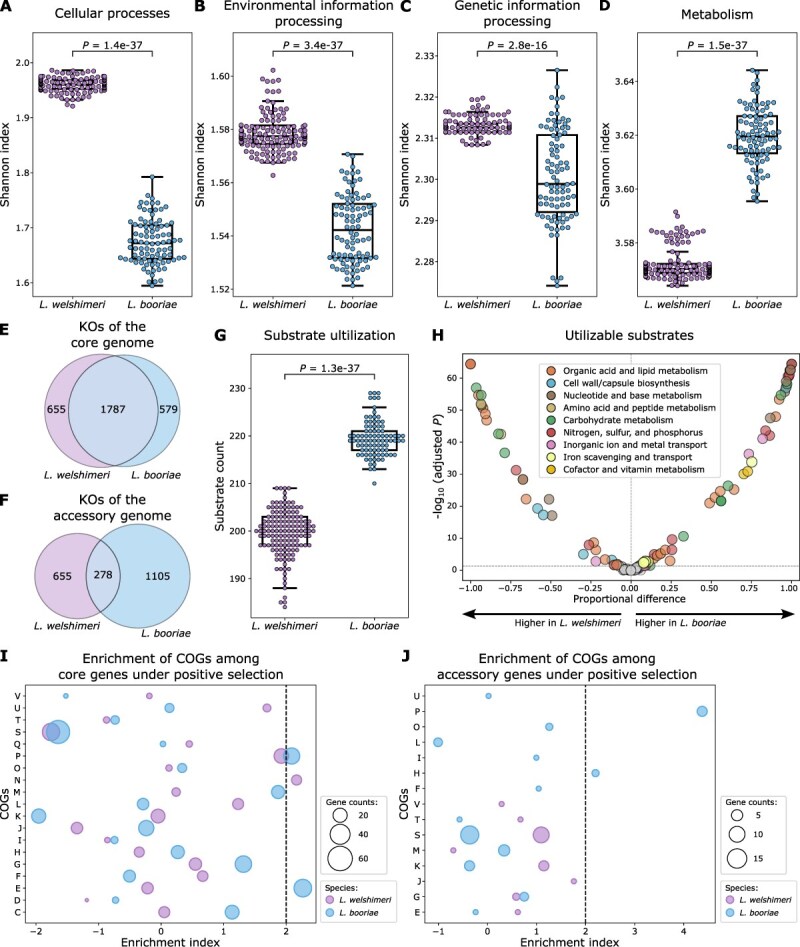
Functional divergence and positive selection in *L. welshimeri* and *L. booriae*; (A–D) Shannon diversity of KEGG pathways compared between *L. welshimeri* and *L. booriae* for (A) cellular processes, (B) environmental information processing, (C) genetic information processing, and (D) metabolism pathways; (E and F) Venn diagrams showing the KO annotations shared between *L. welshimeri* and *L. booriae* for (E) core and (F) accessory genomes; numbers indicate the absolute count of KOs within each sector; (G) total number of utilizable substrates compared between *L. welshimeri* and *L. booriae*; for (A–D) and (G), box plots display the IQR with the median indicated as a line and whiskers extending to 1.5 times the IQR; two-sided MW *U P*-values are annotated; (H) volcano plot showing differences in utilizable substrates between *L. welshimeri* and *L. booriae*, with proportional difference on the *x*-axis and statistical significance (Fisher’s exact *P*) on the *y*-axis; points above the horizontal gray dashed line indicate substrates with adjusted *P* < .05; significant substrates are color-coded by their functions, while non-significant substrates are shown in gray; (I and J) enrichment of COGs among (I) core and (J) accessory genes with evidence of positive selection; an enrichment index >2 (black dashed line) indicates significant enrichment (binomial *P* < .05); circle size is proportional to the number of genes annotated to each COG; COGs abbreviations are as follows: C: energy production and conversion; D: cell cycle control, cell division, and chromosome partitioning; E: amino acid transport and metabolism; F: nucleotide transport and metabolism; G: carbohydrate transport and metabolism; H: coenzyme transport and metabolism; I: lipid transport and metabolism; J: translation, ribosomal structure, and biogenesis; K: transcription; L: replication, recombination, and repair; M: cell wall/membrane/envelope biogenesis; N: cell motility; O: posttranslational modification, protein turnover, and chaperones; P: inorganic ion transport and metabolism; Q: secondary metabolites biosynthesis, transport, and catabolism; T: signal transduction mechanisms; U: intracellular trafficking, secretion, and vesicular transport; V: defense mechanisms.

To assess how the genomic differences between *L. welshimeri* and *L. booriae* are reflected at the functional level, we analyzed KEGG pathway abundance and diversity (see Methods). *L. welshimeri* exhibited significantly greater pathway diversity in cellular processes (MW *U P* = 1.4e-37; Fig. 2A), primarily driven by significantly higher representation of cell motility pathways (|log_2_ fold change| = 3.9, adjusted *P* = 4.0e-37; subgroup 4.5 in Supplementary Figure S3A). *L. welshimeri* also showed significantly higher pathway diversity in (1) environmental information processing (*P* = 3.4e-37; Fig. 2B), such as sensing and regulatory systems (subgroup 3.2 in Supplementary Figure S3B) that include phosphotransferase systems and two-component systems, and (2) genetic information processing (*P* = 2.8e-16; Fig. 2C), including modules involved in DNA replication, recombination, and repair (subgroup 2.4 in Supplementary Figure S3B).

In contrast, *L. booriae* displayed greater diversity in metabolism and overall biological pathways (*P* = 1.5e-37 and 4.5e-14, respectively; Fig. 2D and Supplementary Figure S4). Specifically, 12 metabolism subgroups were significantly different in abundance (adjusted MW *U P* < .05 for all; Supplementary Figure S3A), of which eight were enriched in *L. booriae*, including glycan biosynthesis and metabolism (subgroup 1.7), metabolism of terpenoids and polyketides (1.9), and biosynthesis of other secondary metabolites (1.10). Additionally, 10 of the 12 metabolism subgroups exhibited higher diversity in *L. booriae* (adjusted MW *U P* < .05 for all; Supplementary Figure S3B), including metabolism of terpenoids and polyketides (1.9), glycan biosynthesis and metabolism (1.7), and metabolism of cofactors and vitamins (1.8). Furthermore, analysis of KO overlap between the two species showed that their core genomes shared high similarity in KO annotations, with 73.2% and 75.5% sharing in *L. welshimeri* and *L. booriae*, respectively (Fig. 2E). The metabolic pathways uniquely encoded in the core genomes of each species include biosynthesis of other secondary metabolite in *L. welshimeri*, and the metabolism of other amino acid in *L. booriae* (Supplementary Figure S5). In contrast, accessory genomes showed substantially lower similarity, with only 29.8% and 20.1% sharing KOs for *L. welshimeri* and *L. booriae*, respectively (Fig. 2F). These results suggest that *L. booriae* has greater metabolic diversification and potential compared to *L. welshimeri*, with higher pathway abundance and diversity largely attributable to differences in accessory genes.

To further characterize metabolic capabilities, genome-scale constraint-based metabolic modeling (e.g. flux balance analysis) was performed to predict inferred essential requirements and potentially utilizable substrates for each genome (see Methods). *L. booriae* was predicted to require fewer essential nutrients (Supplementary Figure S6), suggesting enhanced endogenous biosynthetic capacity. Seven essential nutrients (i.e. Ca, K, Mg, Mn, Zn, chloride, and cobalt) were required by all isolates of both species, with riboflavin additionally required by all *L. welshimeri* isolates (Supplementary Table S5). Substrate differential utilization analysis further showed that *L. booriae* was able to utilize a significantly broader range of substrates (*P* = 1.3e-37; Fig. 2G). Specifically, 67 substrates were significantly associated with a greater proportion of *L. booriae* genomes carrying predicted utilization capability, whereas only 34 substrates were associated with *L. welshimeri* (adjusted Fisher exact *P* < .05 for all; Fig. 2H and Supplementary Tables S6 and S7). Specifically, all substrates associated with Fe scavenging and transport, including enterobactin, ferric hydroxamate, ferrienterobactin, and ferrioxamine, were significantly associated or uniquely predicted only in *L. booriae* (Fig. 2H and Supplementary Table S7). Together, these findings suggest substantially broader metabolic capabilities in *L. booriae* compared with *L. welshimeri*, including enhanced potential for Fe metabolism.

Because genome variability in *Listeria* has been linked to positive selection [20], we next identified gene-wide episodic selection in each species using the branch-site unrestricted statistical test for episodic diversification (BUSTED) model. In *L. welshimeri*, 193 core and 31 accessory genes exhibited evidence of positive selection [adjusted likelihood ratio test (LRT) *P* < .05 for all; Supplementary Table S8]. Based on the functional enrichment analysis, core genes of *L. welshimeri* under positive selection were significantly enriched for cell motility (N) functions (binomial *P* < .05; Fig. 2I), including a chemotaxis protein and several flagellar synthesis genes (e.g. *fliH, fliK, flhB, flgC, fliM*). No COGs were significantly enriched among accessory genes under positive selection in this species (Fig. 2J). By contrast, *L. booriae* displayed a broader set of positively selected genes, with 290 core and 64 accessory genes under positive selection (adjusted LRT *P* < .05 for all; Supplementary Table S8). Both core and accessory genes under positive selection were enriched for inorganic ion transport and metabolism (P; Fig. 2I and J), including genes associated with Fe acquisition such as enterochelin esterase and a related enzyme, as well as Fe ATP-binding cassette (ABC) transporter substrate-binding protein. Core and accessory genes under positive selection were also enriched for amino acid transport and metabolism (E; Fig. 2I) and coenzyme transport and metabolism (H; Fig. 2J), respectively. These patterns suggest that *L. booriae* adapts to environmental conditions through enhanced metabolic versatility, including improved Fe acquisition, whereas *L. welshimeri* may gain fitness advantages from cell motility adaptation in soils.

### *Listeria booriae* shows strong genomic associations with abiotic environmental conditions and bacterial community composition

As our analyses identified strong evidence of clade-level endemism and environmental adaptation associated with metabolic versatility in *L. booriae*, we hypothesized that its genome content is strongly shaped by local environmental conditions. To test this hypothesis, we compared the abiotic conditions and bacterial communities of *L. welshimeri* and *L. booriae* habitats and assessed their correlations with gene richness. A total of 21 abiotic factors and 15 bacterial phyla were found to be significantly different between their habitats (adjusted MW *U P* < .05 for all). *L. welshimeri* was primarily isolated from sites with significantly higher minimum temperature, elevation, pH, and concentration of certain soil macronutrients (K, Mg, Ca, P), greater coverage of pasture, wetland, and cropland in surrounding areas (Supplementary Figure S7A), and higher relative abundances of Proteobacteria, Planctomycetes, Elusimicrobia, and Chlamydiae (Supplementary Figure S7B). In contrast, *L. booriae* occurred in sites with significantly higher precipitation, greater forest and shrubland coverage in surrounding areas, elevated soil concentrations of Fe, Al, Zn, Mn, and Cu (Supplementary Figure S7A), and were enriched with Actinobacteria, Chloroflexi, Bacteroidetes, Firmicutes, and Fibrobacteres (Supplementary Figure S7B). Consistent with these results, both MDS plots based on Euclidian distance of 34 abiotic variables and bacterial community beta diversity indicated by weighted UniFrac distances reveal significant clustering of samples by species (PERMANOVA *P* = .001 for both, pseudo-*F* = 18.8% and 32.6%, respectively; Supplementary Figure S8A and B), suggesting that *L. welshimeri* and *L. booriae* occupied distinct environmental abiotic and biotic spaces.

**Figure 3:**
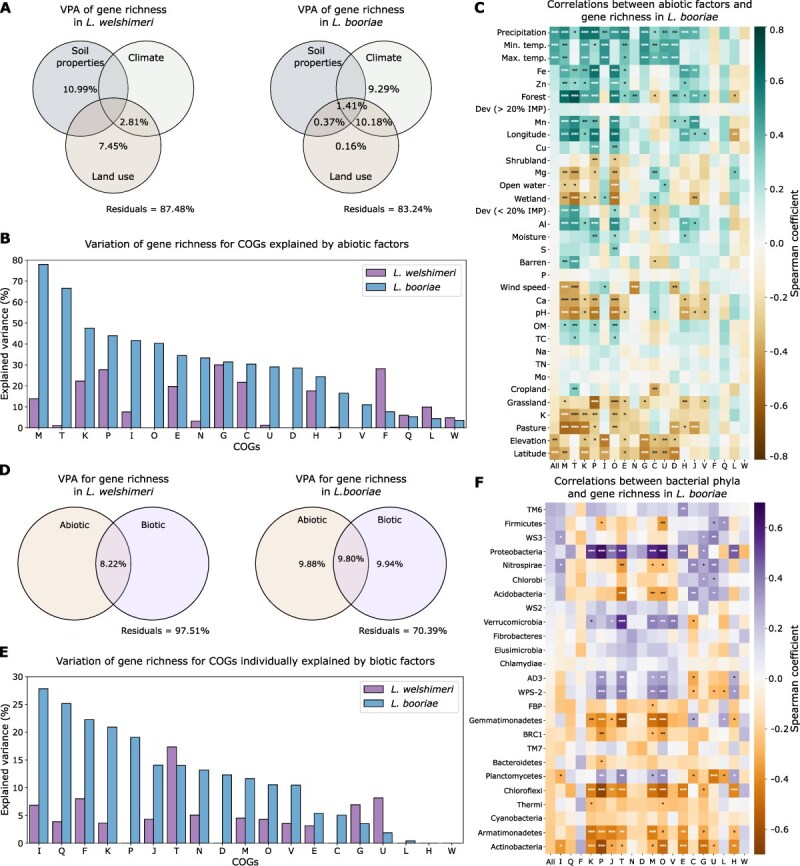
Associations between gene richness in *L. welshimeri* and *L. booriae* and abiotic environmental factors and bacterial community composition; (A) Venn diagrams from VPA showing the proportion of overall gene richness variation explained by soil properties, climate, and surrounding land use for *L. welshimeri* and *L. booriae*; (B) variation in gene richness of COG categories explained by abiotic factors based on VPA; (C) Spearman’s correlations between abiotic factors and gene richness, both overall and for COGs, in *L. booriae*; abiotic factors are ordered by the descending correlation coefficients of overall (“all”) gene richness; positive and negative correlations are shown in green and brown, respectively; (D) Venn diagrams from VPA showing the proportion of gene richness variation explained by abiotic and biotic factors (i.e. surrounding bacterial phyla) for *L. welshimeri* and *L. booriae*; for (A) and (D), only positive fractions of explained variance are shown; negative fractions were omitted, and therefore the total explained variance and the residual does not sum to 100%; (E) variation in gene richness of COG categories individually explained by biotic factors based on VPA; (F) Spearman’s correlations between the relative abundance of bacterial phyla and gene richness, both overall and for COGs, in *L. booriae*; bacterial phyla are ordered by the descending correlation coefficients of overall (“all”) gene richness; TM6, WS3, WS2, AD3, WPS-2, FBP, BRC1, and TM7 represent candidate bacterial phyla that remain uncultured under laboratory conditions; positive and negative correlations are shown in purple and orange, respectively; for (C) and (F), significance levels are indicated as “^*^,” “^**^,” and “^***^” for adjusted *P* < .05, < .01, and < .001, respectively.

For the genomic associations with abiotic factors, VPA using VIF-selected variables (see Methods) showed that overall abiotic factors explained more gene richness variation in *L. booriae* (16.8%) than in *L. welshimeri* (12.5%; Fig. 3A). Climate and soil properties exhibited the largest individual contributions in gene richness in *L. booriae* (9.3%) and *L. welshimeri* (11.0%), respectively (Fig. 3A). When stratified by COGs, abiotic factors explained more variation in *L. booriae* than in *L. welshimeri* for 15 of 19 COGs (78.9%), with the most variation explained for cell membrane biogenesis (M; 77.9%), followed by signal transduction mechanism (T; 66.5%) and transcription (K; 47.5%; Fig. 3B). Consistent with the VPA results, Spearman correlation analyses showed that gene richness exhibited stronger associations with individual abiotic factors in *L. booriae*. Overall gene richness in *L. booriae* was positively associated with precipitation and temperature, and negatively with latitude and elevation (adjusted *P* < .05 for all; Fig. 3C), whereas no significant association was observed for *L. welshimeri* (Supplementary Figure S9). Across COGs, an average of 10 abiotic factors were significantly associated with gene richness in *L. booriae*, while *L. welshimeri* only had five factors (Fig. 3C and Supplementary Figure S9). Notably, precipitation and Fe showed strong correlations (|Spearman’s *ρ*| > 0.5) with four COGs, including the three COGs, inorganic ion (P), amino acid (E), coenzyme (H) transport and metabolism, targeted by positive selection in *L. booriae* (Fig. 2I and J). These findings suggest that abiotic factors, particularly precipitation and Fe, play an important role in shaping gene content in *L. booriae.*

In addition, VPA using VIF-selected variables showed that abiotic factors and surrounding bacterial phyla together explained substantially more variation in gene richness in *L. booriae* (29.6%) than *L. welshimeri* (2.5%; Fig. 3D). Surrounding bacterial phyla individually accounted for 9.9% of the variation in *L. booriae*, whereas they had minimal explanatory power in *L. welshimeri* (Fig. 3D). In *L. welshimeri*, the total explained variance for overall gene richness decreased from 12.5% (abiotic factors only) to 2.5% after including bacterial phyla, suggesting that bacterial phyla explained little additional variance and that their inclusion resulted in a penalty for increased model complexity [42]. When stratified by COGs, surrounding bacterial phyla individually explained more variation in *L. booriae* than in *L. welshimeri* for 14 of 19 COGs (73.7%), with the most variation explained for lipid transport and metabolism (I; 27.8%), followed by secondary metabolites biosynthesis, transport and catabolism (Q; 25.1%) and nucleotide transport and metabolism (F; 22.3%; Fig. 3E). Concordantly, Spearman correlation analyses indicated that gene richness of *L. booriae* exhibited stronger associations with the relative abundance of bacterial phyla. Although overall gene richness in *L. booriae* showed no significant correlations with any individual bacterial phylum (Fig. 3F), COG-level analyses revealed substantially more significant associations in this species, averaging six correlations per phylum compared with one in *L. welshimeri* (Fig. 3F and Supplementary Figure S10). In *L. booriae*, Actinobacteria and Proteobacteria showed the highest number of strong correlations with COG-specific gene richness (|Spearman’s *ρ*| > 0.5; Fig. 3F). Genes involved in inorganic ion transport and metabolism (P), which exhibited signatures of positive selection (Fig. 2I and J), were associated with both phyla (Fig. 3F). Consistent with phylum-level observations, the number of genera and species significantly associated with gene richness was substantially higher in *L. booriae* than in *L. welshimeri*, including 72 versus 35 genera and 16 versus 11 species, respectively (adjusted Spearman’s *P* < .05 for all; Supplementary Tables S9 and S10). Specifically, the species uniquely associated with *L. booriae* were predominantly Actinobacteria species (e.g. *Nocardioides halotolerans, Nocardioides pyridinolyticus*) and Proteobacteria species (e.g. *Burkholderia bryophila*; Supplementary Figure S11). Notably, gene richness for inorganic ion transport and metabolism (P) showed the greatest number of significant associations at both the genus and species levels among all COGs (Supplementary Figure S12). Even though more bacterial phyla, genera, and species were significantly associated with gene richness in *L. booriae*, samples positive for this species exhibited significantly lower bacterial diversity and evenness than *L. welshimeri* (MW *U P* = 7.9e-04 and 9.7e-07, respectively; Supplementary Figure S13). These results suggest that *L. booriae* persists in lower-diversity communities with potentially stronger biotic interactions, which may facilitate cross-feeding dynamics within comparatively simplified microbial networks. Collectively, these findings suggest that like abiotic factors, bacterial community composition, particularly Actinobacteria and Proteobacteria, is important to the gene content, especially that involved in inorganic ion transport and metabolism, in *L. booriae*.

### Wildlife, particularly regional-terrestrial birds, appears to facilitate long-distance dispersal of *Listeria welshimeri* in soil

**Figure 4:**
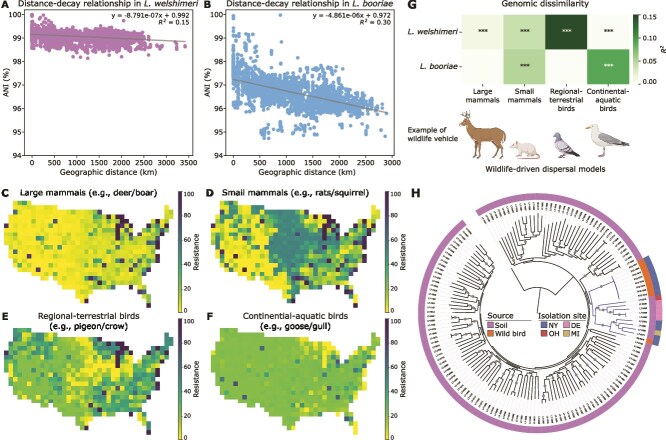
Dispersal patterns of *L. welshimeri* and *L. booriae* and evidence of wild birds as a dispersal vehicle for *L. welshimeri* in soil; (A and B) distance–decay relationships for (A) *L. welshimeri* and (B) *L. booriae*, inferred by the linear regression for genetic similarities, measured by ANI, and geographic distances; a steeper negative slope with a higher *R*^2^ indicates a stronger distance–decay relationship; (C–F) landscape resistance surfaces representing wildlife connectivity for (C) large mammals, (D) small mammals, (E) regional-terrestrial birds, and (F) continental-aquatic birds; to facilitate visualization at the continental scale, resistance surfaces were down sampled by a factor of 50; the color gradient from bright yellow to dark blue indicates increasing landscape resistance, ranging from optimal dispersal corridors (1) to absolute barriers (100); (G) linear regressions between genomic difference (1 – ANI) and resistance distance for each wildlife connectivity model; the color gradient from light to dark green indicates increasing *R*^2^ values; significance levels for linear slopes are indicated as “^*^,” “^**^,” and “^***^” for *P* < .05, < .01, and < .001, respectively; (H) maximum likelihood phylogenetic tree based on core SNPs of 141 soil *L. welshimeri* isolates (purple) and nine wild bird isolates (orange); the tree was constructed using 1000 bootstrap replicates, and bootstrap values >80% are indicated by gray circles on the corresponding branches; the tree was rooted by midpoint; branches belonging to the monophyletic clade that contains both soil and wild bird isolates are highlighted in blue, and the collection sites for isolates within this clade are annotated; NY, DE, OH, and MI denote the states of New York, Delaware, Ohio, and Michigan, respectively.

As *L. welshimeri* had minimal geographically confined clades and signatures of positive selection on cell motility genes, and showed relatively weaker associations with environmental conditions than *L. booriae*, we hypothesized that it frequently disperses across geographic locations. To test this hypothesis, we analyzed the distance–decay relationships for both species. The slope of the linear relationship between ANI and geographic distance was 5.5-fold shallower in *L. welshimeri* (*R*^2^ = 0.15, slope = −8.79e-07; Fig. 4A) compared with *L. booriae* (*R*^2^ = 0.30, slope = −4.86e-06; Fig. 4B), and gene content similarity showed a similar pattern (3.4-fold shallower slopes; *L. welshimeri*: *R*^2^ = 0.05, slope = −7.98e-06; *L. booriae*: *R*^2^ = 0.22, slope = −2.73e-05; Supplementary Figure S14). These results showed that *L. welshimeri* exhibited a much weaker distance–decay relationship, suggesting more frequent dispersal across geographic locations.

The reported motility speeds for *Listeria* were ~0.09 μm s^–1^ in cytoplasm [43, 44] and ~10 μm s^–1^ on a non-charged microscope slide [45]. Combined with the physical constraints of soil environments [46, 47], it is unlikely that long-distance dispersal is achieved by motility alone. Thus, we hypothesized that mobile hosts, such as wildlife, play a role. To test this hypothesis, we conducted ecological connectivity modeling for four potential *Listeria* dispersal vectors: small mammals, large mammals, regional-terrestrial birds, and continental-aquatic birds (see Methods). First, we generated continental-scale resistance surfaces to characterize wildlife movement pathways. Mammalian pathways were characterized by strong fragmentation associated with human-modified infrastructure and barren lands (Fig. 4C and D), whereas avian models demonstrated specialized spatial routing (Fig. 4E and F). Optimal corridors for regional-terrestrial birds were highly aggregated within agricultural and developed matrices (Fig. 4E), whereas continental-aquatic pathways were highly constrained to continental hydrological networks and wetland staging areas (Fig. 4F). Ecological connectivity was subsequently modeled (Supplementary Table S11). Results showed that resistance surface was significantly associated with the genomic dissimilarity of *L. welshimeri* across locations for all four wildlife groups (*P* < .001 for all; Fig. 4G). The regional-terrestrial bird connectivity model explained the largest proportion of the genetic dissimilarity in *L. welshimeri* (*R*^2^ = 0.143), followed by small mammals (*R*^2^ = 0.041), large mammals (*R*^2^ = 0.012), and continental-aquatic birds (*R*^2^ = 0.001). In contrast, only the resistance surface for continental-aquatic birds and small mammals were significantly associated with the genomic dissimilarity of *L. booriae* (*R*^2^ = 0.090 and 0.051, respectively, *P* < .001 for both; Fig. 4G). These results suggest the important role of wildlife, particularly regional-terrestrial birds, in facilitating the long-distance dispersal of *L. welshimeri* across soil ecosystems.

Consistent with this inference, we recovered nine *L. welshimeri* isolates from wild regional-terrestrial birds (i.e. grosbeak and pigeon) in New York and performed WGS followed by comparative genomic analyses for soil and wild bird isolates. Core-SNP phylogenetic analysis revealed that the wild bird isolates clustered within a monophyletic clade alongside nine soil isolates from nearby regions, including Ohio, Delaware, New York, and Michigan (Fig. 4H). As eight of the nine wild bird isolates formed a tight clade with minimal SNPs, suggesting they represent a single strain, R12-1511 was selected as the representative strain. The representative wild bird and soil isolates forming a monophyletic clade shared high ANI (mean: 99.3%, range: 99.2%–99.5%), with no significant difference observed among within-soil, soil-wild bird, and within-wild bird groups (KW *P* = .065; Supplementary Figure S15). Comparisons of genome size, gene richness, and GC content also revealed no significant differences between soil and wild bird isolates within the monophyletic clade (MW *U P* = .178, .178, and .555, respectively; Supplementary Figure S15). These results show close genomic relatedness between *L. welshimeri* isolates from soil and wild regional-terrestrial birds, which suggests potential movement of strains between these habitats. This finding further supports wild regional-terrestrial birds as a plausible mechanism for long-distance dispersal and reduced geographic structuring in *L. welshimeri*.

## Discussion

**Figure 5:**
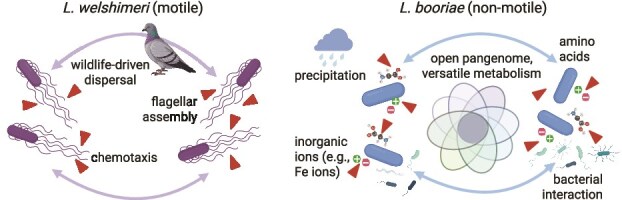
Ecological and evolutionary strategies associated with the broad geographic ranges in *L. welshimeri* and *L. booriae*; triangles mark functions targeted by positive selection: cell motility in *L. welshimeri* and inorganic ion, amino acid, and coenzyme transport and metabolism in *L. booriae*; created in BioRender.

Here, we show strong evidence that motile and non-motile cosmopolitan *Listeria* species adopt distinct genomic foundations and ecological and evolutionary strategies to achieve broad geographic ranges in soil ecosystems (Fig. 5). *L. welshimeri,* which is a motile species, appears to achieve a widespread distribution through effective long-distance dispersal potentially mediated by wildlife vectors, particularly regional-terrestrial birds. In contrast, *L. booriae*, a non-motile species, likely attains a broad geographic range through effective environmental adaptation, particularly those shaped by Fe concentration and precipitation, by maintaining an open pangenome characterized by abundant and diverse metabolic pathways and broad substrate utilization capacity, and strong associations with abiotic and surrounding bacterial community composition.

Flagellar motility and chemotaxis are central to bacterial nutrient sensing and the exploitation of heterogeneous environments [12, 16, 48–50]. In soil, where nutrients are highly patchy and often transient [47], the ability to detect and move toward favorable chemical gradients confers a strong fitness advantage. Flagella-driven motility allows bacteria to navigate toward nutrient hotspots and away from unfavorable conditions, facilitating rapid colonization of resource-rich microenvironments [51]. In *L. welshimeri*, we observed that flagellar and chemotaxis genes were targeted by positive selection, which may have contributed to its niche expansion. Consistent with this finding, laboratory and evolutionary experiments have shown that, under selective pressure, *Escherichia coli* can evolve enhanced swimming velocity and altered tumbling rates, improving habitat colonization and nutrient acquisition [52]. In addition, the increased diversity of pathways in cellular processes and environmental information processing observed in *L. welshimeri* may reflect an expanded repertoire of sensing and regulatory systems (e.g. the phosphotransferase system and two-component systems), and together with the presence of flagellar assembly pathways, enhances its ability to detect and respond to diverse external stimuli, potentially benefiting niche expansion.

*Listeria welshimeri* also appears to leverage flagella to broaden its geographic range through enhanced host attachment and colonization, which may facilitate movement across landscapes. Beyond motility, flagellar genes can facilitate adhesion, host attachment, and colonization [49, 53, 54]. For example, in *Clostridioides difficile*, flagellin is essential for initial attachment to mucosal surfaces [53], whereas in *Helicobacter pylori*, deletion of *fliK* reduces adhesion to gastric mucosal cells [54]. Both *H. pylori* and *Campylobacter jejuni* regulate flagellar biosynthesis and chemotaxis to penetrate viscous milieus, such as gastrointestinal mucus, facilitating host colonization [49]. In *Listeria*, flagella similarly enhance host cell adhesion and invasion by increasing the bacterium-host contact frequency and facilitating early attachment [55–57]. Such host-associated interactions may help bacteria overcome geographic barriers and contribute to movement across large spatial scales [58]. In this study, ecological connectivity modeling identified wild regional-terrestrial birds as potentially important vectors for *L. welshimeri* to disperse across the United States. This finding was further supported by the close phylogenetic relationships observed between *L. welshimeri* isolates from soil and wild birds (grosbeaks and pigeons). Wild regional-terrestrial birds, such as starlings and pigeons, have been reported as carriers and dispersal vectors of *Listeria* [23, 59] and avian-mediated dispersal has been shown to shape the biogeographic patterns of *L. monocytogenes* [59] and *E. coli* [60]. In addition, other passive transport routes, including anthropogenic, hydrological, and environmental pathways, may represent alternative or complementary explanations for *L. welshimeri* dispersal, as sites positive for *L. welshimeri* were associated with significantly higher proportions of surrounding cropland, wetland, and open water land use (Supplementary Figure S16A).

By nature, non-motile bacteria face intrinsic constraints on niche expansion because they lack motility. However, based on our systematic, large-scale survey, the non-motile species *L. booriae* was previously found to be highly ecologically successful in soil, occupying a broad geographic range similar to that of *L. welshimeri* [20]. In the present study, we found that the broad geographic range of *L. booriae* is associated with extensive metabolic versatility enabled by a large, open pangenome, characterized by a diverse repertoire of genes involved in metabolic pathways. This genomic expansion likely reduces the requirement of essential nutrients and facilitates the exploitation of diverse ecological resources as observed in this study, potentially enabling broad niche colonization and adaptation to new environments. Also, genes involved in metabolic functions, spanning both the core and accessory genome, show strong signatures of positive selection in *L. booriae*. Major adaptive traits included inorganic ion transport, amino acid and coenzyme metabolism, and protein biosynthesis. These processes are essential for nutrient acquisition, enzymatic activity, and cellular maintenance [61]. Enhanced metabolic efficiency, flexibility, and diversification likely enable *L. booriae* to thrive across nutrient-variable environments, possibly compensating for its lack of motility. Unlike *L. welshimeri*, we concluded that wildlife-driven long-distance dispersal is unlikely to be the primary mechanism underlying the broad geographic range of *L. booriae*. While the genomic similarity of *L. booriae* was significantly associated with the movement pathways for small mammals and continental-aquatic birds, the explained variable was not large (<10%). Also, given the disadvantage that *L. booriae* cannot actively locate and access favorable host-associated niches to colonize wildlife and dispersal limitation suggested by the distance-decay analysis (Fig. 4B), together with the fact that, to our knowledge, *L. booriae* has never been isolated from wildlife, wildlife colonization does not appear to be an effective strategy for this species to disperse. Although passive transmission, such as anthropogenic and environmental transport, may contribute to *L. booriae* dispersal, as suggested by higher proportions of surrounding cropland and developed open-space land use with <20% IMP cover at *L. booriae*-positive sites (Supplementary Figure S16B), such processes are likely restricted to regional scales.

Our data suggest that the abiotic environmental pressures playing a vital role in shaping the pangenome variation and metabolic versatility of *L. booriae* are Fe concentration and precipitation. *L. booriae* appears to respond to these environmental fluctuations through the retention or positive selection of genes involved in inorganic ion, coenzyme, and amino acid transport and metabolism. Notably, all substrates associated with Fe scavenging and transport were significantly associated or uniquely predicted in *L. booriae* (Fig. 2H). Consistent with this finding, frequent positive selection was detected among genes associated with Fe acquisition, such as enterochelin esterase and related enzyme, as well as Fe ABC transporter substrate-binding protein (Supplementary Table S8). Along with its occurrence in sites with significantly higher Fe concentrations (Supplementary Figure S7A) and the strong positive association between Fe and gene richness, including those involved in inorganic ion transport and metabolism (Fig. 3C), these results are consistent with metabolic diversification linked to adaptation to local Fe-related environmental conditions. Similar adaptive strategies have been reported in rhizosphere communities, where Fe metabolism genes are enriched in Actinobacteria under drought conditions that reduce Fe bioavailability [62]. In addition, it has been reported that soil microbial metabolic genes are influenced by climatic variation [62–64]. Precipitation, in particular, functions as a dominant selective filter by alternately promoting the solubilization of essential mineral ions and nutrients, such as Fe and amino acids, and driving their depletion [65, 66].

In addition to abiotic factors, we identified strong associations between bacterial community composition, particularly the relative abundance of Actinobacteria (e.g. *N. halotolerans, N. pyridinolyticus*) and Proteobacteria (e.g. *B. bryophila*), and richness of genes, particularly for genes involved in inorganic ion transport and metabolism, in *L. booriae*. Members of these phyla are known to possess diverse systems for inorganic ion acquisition and nutrient transformation [67, 68]. For example, *Nocardioides* species, such as *N. simplex*, harbor desferrioxamine gene clusters involved in the secretion of hydroxamate siderophores, providing stable, bioavailable Fe [69]. The significant negative correlations between *N. halotolerans* and *N. pyridinolyticus* and inorganic ion transport and metabolism genes in *L. booriae* (Supplementary Table S10) suggests that *Nocardioides* may provide a stable source of bioavailable Fe. Under such conditions, *L. booriae* may rely on interspecies cross-feeding interactions, reducing the need to maintain auxiliary ion transport genes. In contrast, *B. bryophila* secretes extracellular siderophores to scavenge Fe under Fe-depleted conditions [70]. The positive correlation between *B. bryophila* and inorganic ion transport and metabolism genes in *L. booriae* (Supplementary Table S10) suggests that *L. booriae* may retain or acquire auxiliary ion transport systems to compete under Fe-limited conditions. Of note, inference of metabolic interactions within microbial communities is limited by the resolution of 16S rRNA gene amplicon sequencing. Future metagenomic analyses and co-culture experiments will be necessary to validate these ecological relationships and reconstruct pathways involved in community-mediated nutrient provisioning and inorganic ion acquisition that may influence *Listeria* ecology.

Together, our findings delineate distinct genomic foundations and ecological and evolutionary strategies associated with the broad geographic ranges of cosmopolitan motile and non-motile *Listeria* species, refining our understanding of how bacteria with contrasting lifestyles achieve comparably broad ecological distributions and resilience to environmental pressures. Our study highlights often-overlooked mechanisms that enable non-motile bacteria to survive and persist in soil ecosystems. These insights have important implications for controlling and minimizing environmental transmission of motile and non-motile pathogens. Given the importance of *Listeria* to food safety, our findings support more targeted, species-specific surveillance and intervention strategies that account for key physiological traits, particularly motility, throughout food production, processing, and storage.

## Supplementary Material

Supplementary_material_wrag158

## Data Availability

Whole-genome sequencing data for 141 *L. welshimeri* and 90 *L. booriae* soil isolates used in this study have been submitted to the NCBI BioProject database under accession number PRJNA561882, along with associated environmental metadata, previously published in Liao et al. [20]. The 16S rRNA gene amplicon sequencing data analyzed in this study are available under BioProject accession number PRJNA749132, as published in Liao et al. [24]. Trimmed paired-end reads for the nine *L. welshimeri* wild bird isolates used in this study have been deposited in the NCBI Sequence Read Archive, and their assembled genomes are available in NCBI GenBank under the accession numbers listed in Supplementary Table S1. All data needed to evaluate the conclusions in the paper are present in the paper and/or the Supplementary Information.

## Code availability

Code to replicate all analyses is publicly available at https://github.com/leaph-lab/Listeria_motility_eco_MS.
