# Supplementary material for: Motile and non-motile Listeria species adopt distinct ecological and evolutionary strategies to achieve broad geographic ranges across soil ecosystems
Source: ISME J. 2026 Jun 19;20(1):wrag158. doi: 10.1093/ismejo/wrag158 (PMC13374856; doi:10.1093/ismejo/wrag158)
Supplement: Supplementary_material_wrag158 [file supplementary_material_wrag158.zip › Supplementary_Info_final_wrag158.docx]

Supplementary Information

**Motile and non-motile *Listeria* species adopt distinct ecological and evolutionary strategies to achieve broad geographic ranges across soil ecosystems.**

**Ying-Xian Goh, Shannon Hepp, Kevin J. Cummings, Martin Wiedmann, Jingqiu Liao**

**Supplementary Methods**

**WGS data preprocessing for *L. welshimeri* wild bird isolates**

Sequencing adapters and low-quality reads were trimmed using fastp v0.23.4 [1], and cleaned reads were assembled de novo with SPAdes v4.0.0 [2]. Contigs <500 bp were removed. Assembly quality was assessed using QUAST v5.2.0 [3]; coverage was assessed using BBmap v39.01 [4] and Samtools v1.17 [5]; genome completeness and contamination were assessed using CheckM2 v1.0.2 [6]; and taxonomic assignment was assessed using Kraken2 v2.1.3 [7]. All assemblies met the following quality criteria: <300 contigs, N50 >50,000 bp, average coverage >30×, completeness >90%, contamination <5%, and species identity confirmed as *L. welshimeri*. Genes of wild bird isolates were predicted using Prodigal v2.6.3 [8], and ortholog genes present in both soil and wild bird isolates were identified using MMseqs2 [9].

**Positive selection**

Core and accessory genes of each species were screened for positive selection using the branch-site unrestricted statistical test for episodic diversification (BUSTED) model implemented in Hypothesis Testing Using Phylogenies (HyPhy) [10]. Genes were included if they contained at least three non-identical sequences and exhibited one or more non-synonymous substitutions. Evidence of positive selection was evaluated using a likelihood ratio test (LRT) comparing unconstrained and null models, with significance assessed via a χ^2^ test followed by BH-FDR correction. Homologous recombination was also assessed for both core and accessory genes using the Recombination Detection Program v4 (RDP4) [11].

**Characteristics of resistance surfaces for different wildlife dispersal vectors**

To evaluate the impact of landscape heterogeneity on *Listeria* dispersal, landscape resistance surfaces were generated for four distinct ecological models representing common wildlife vectors: large mammals, small mammals, regional-terrestrial birds, and continental-aquatic birds (**Supplementary Table 4**). Resistance values were assigned to 2024 National Land Cover Database (NLCD) land cover classes [12] at 30 m spatial grain based on species-specific behavioral ecology, habitat preferences, and movement capabilities. A standard resistance scale was utilized across all models: optimal habitat or primary dispersal corridors were assigned a resistance value of 1, neutral matrix environments that permit movement but are non-optimal were assigned intermediate resistance values (20-50), and absolute geographic or anthropogenic barriers were assigned high resistance values (80-100).

For large mammals, the surface was parameterized to reflect the movement ecology of wide-ranging species, such as deer and boar. These animals prioritize landscapes that offer a balance of adequate forage and structural cover. Thus, optimal resistance (resistance = 1) was assigned to deciduous and evergreen forests, shrublands, grasslands, and agricultural matrices [13, 14]. Open water and high-density urban infrastructure represent severe barriers (resistance = 100) due to physical movement limitations and high mortality risks, such as vehicle collisions, while barren lands and wetlands serve as permeable but non-preferred transit environments (resistance = 20-50). For small mammals, such as mice and rats, dispersal is heavily constrained by predation risk. Optimal dispersal corridors (resistance = 1) were explicitly restricted to environments providing dense structural cover, such as continuous forests and woody wetlands [15]. Open herbaceous areas were designated as a neutral matrix (resistance = 50). Conversely, open water, barren land, and all developed classes were coded as severe barriers (resistance = 80-100); these environments lack necessary protective cover and are associated with extreme mortality rates during road crossings [16, 17].

The regional-terrestrial bird model represents species characterized by land-based, regional dispersal and an affinity for human-modified landscapes, including synanthropic and agricultural-associated species such as pigeons, rooks, and crows. The model assumes a storong preference for human-modified habitats. Therefore, developed open spaces, pastures, and cultivated crops were classified as optimal dispersal corridors (resistance = 1) due to the high availability of anthropogenic food sources and favorable foraging conditions [18–20]. Conversely, deep, continuous forest tracts were assigned a higher resistance (resistance = 50) to reflect their position outside the focal human-associated niche, while open water serves as an absolute barrier (resistance = 100) for these non-aquatic species. The continental-aquatic bird model reflects the long-distance, water-dependent aerial dispersal networks typical of waterfowl and migratory shorebirds, including geese and gulls. Hydrological features and emergent wetlands, which serve as critical staging and stopover sites for resting and refueling, were assigned optimal resistance (resistance = 1) [21, 22]. The majority of terrestrial landscapes were classified as a neutral flyover matrix (resistance = 20) to represent high-altitude movement where ground-level habitat is less influential. High-intensity urban centers were modeled as significant barriers (resistance = 80-100) to account for active avoidance behaviors, light-pollution disorientation, and the high collision risks associated with dense urban infrastructure [23, 24].

**Supplementary Figures**

Supplementary Figure 1. Geographic distribution of the major clades of *L. welshimeri* and *L. booriae*. (A) Pairwise geographic distances between isolates within major clade of each species. Major clades were identified based on the phylogenetic trees shown in Fig.1 C-D and are color-coded, with “Lw” and “Lb” indicating *L. welshimeri* and *L. booriae*, respectively. White dots indicate the mean pairwise distance within each major clade. (B) Mean pairwise geographic distance between isolates across major clades compared between *L. welshimeri* and *L. booriae*. Two-sided Mann-Whitney (MW) *U P* value is annotated. For (A) and (B), box plots show the interquartile range (IQR), with the median indicated by a horizontal line and whiskers extending to 1.5 times IQR.

Supplementary Figure 2. Pangenome openness and genome size of *L. welshimeri* and *L. booriae*. (A) Accumulation curves of the core genome and pangenome of *L. welshimeri* and *L. booriae*. The lower curves represent core genome accumulation, whereas the upper curves represent pangenome accumulation. These curves were adapted from Extended Data Fig. 7 in Liao et al (2021) [25]. (B) Genome sizes of isolates compared between *L. welshimeri* and *L. booriae*. Box plots show the IQR, with the median indicated by a horizontal line and whiskers extending to 1.5 times IQR. Two-sided MW *U P* value is annotated.

**Supplementary Figure 3. KEGG pathway subgroup abundance and diversity in *L. welshimeri* and *L. booriae*. (A-B)** Volcano plot showing differences in **(A)** KEGG pathway subgroup abundance and **(B)** KEGG pathway subgroup diversity between *L. welshimeri* and *L. booriae*, with fold change on the x-axis and significance (two-sided MW *U P*) on the y-axis. Points above the horizontal gray dashed line indicate adjusted *P* < 0.05. Significant subgroups are color-coded by major KEGG pathway category, whereas non-significant subgroups are shown in gray. Abbreviations for KEGG pathway subgroups are provided in **Supplementary Table 2**.

**Supplementary Figure 4. Shannon-Wiener diversity of overall biological pathways compared between *L. welshimeri* and *L. booriae*.** Box plots show the IQR, with the median indicated by a horizontal line and whiskers extending to 1.5 times IQR. Two-sided MW *U P* value is annotated.

**Supplementary Figure 5.** Species-specific metabolic pathways encoded by the core genomes of *L. welshimeri* (purple) and *L. booriae* (blue). Shared pathways are shown in gray.

**Supplementary Figure 6.** Distribution of essential nutrient requirements predicted from genome-scale metabolic models in *L. welshimeri* (purple) and *L. booriae* (blue).

**Supplementary Figure 7. Individual abiotic and biotic environmental factors compared between *L. welshimeri* and *L. booriae*. (A-B)** Volcano plots showing fold change (x-axis) versus statistical significance (y-axis, adjusted two-sided MW *U* test) for **(A)** abiotic factors and **(B)** relative abundance of bacterial phyla. Points above the gray dashed line indicate adjusted *P* < 0.05 and are color-coded by abiotic factor group in (**A**) and by the species with a higher relative abundance in (**B**), whereas non-significant abiotic factors and bacterial phyla are shown in gray. Abbreviations of abiotic factors are described in Methods. WPS-2, AD3, TM6, and BRC1 represent candidate bacterial phyla that remain uncultured under laboratory conditions.

**Supplementary Figure 8. Overall abiotic and biotic environmental conditions compared between *L. welshimeri* and *L. booriae****.* **(A-B)** Multidimensional scaling (MDS) analysis based on **(A)** Euclidean distances of abiotic factors and **(B)** weighted UniFrac distances of bacterial community composition derived from OTUs. Points represent isolates and are color-coded by species. Ellipses indicate two standard deviations from the mean. PERMANOVA *P* < 0.05 denotes significant clustering by species.

**Supplementary Figure 9. Associations between gene richness in *L. welshimeri* and abiotic environmental factors.** Spearman’s correlations between abiotic factors and gene richness, both overall and for COGs, in *L. welshimeri*. Abiotic factors are ordered by the descending correlation coefficients of overall (“All”) gene richness. Positive and negative correlations are shown in green and brown, respectively. Abbreviations of COGs and abiotic factors are described in figure legend of **Fig. 2** and in the Methods, respectively.

**Supplementary Figure 10. Associations between gene richness in *L. welshimeri* and bacterial community composition.** Spearman’s correlations between the relative abundance of bacterial phyla and gene richness, both overall and for COGs, in *L. welshimeri*. Bacterial phyla are ordered by the descending correlation coefficients of overall (“All”) gene richness. Positive and negative correlations are shown in purple and orange, respectively. Abbreviation of COGs is described in figure legend of **Fig. 2**. TM6, WS3, WS2, AD3, WPS-2, FBP, BRC1, and TM7 represent candidate bacterial phyla that remain uncultured under laboratory conditions.

**Supplementary Figure 11.** Sankey diagrams showing bacterial genera (left) and species (right) significantly associated with gene richness by COGs in *L. booriae* and their corresponding phyla. Box height and line thickness represent the number of significant associations between gene richness and genera/species within a given phylum. Detailed results of the Spearman correlation analyses, including correlation coefficients and raw and adjusted *P* values, are provided in **Supplementary Tables 9** and **10**.

**Supplementary Figure 12.** Number of genus (blue) and species (orange) significantly associated with gene richness by COGs in *L. booriae*. Detailed results of the Spearman correlation analyses, including coefficients and raw and adjusted *P* values, are provided in **Supplementary Tables 9** and **10**.

**Supplementary Figure 13. (A)** Shannon-Wiener diversity and **(B)** Pielou’s evenness at sites where *L. welshimeri* and *L. booriae* were isolated. Box plots show the IQR, with the median indicated by a horizontal line and whiskers extending to 1.5 times IQR. Two-sided MW *U P* value is annotated.

**Supplementary Figure 14. Dispersal patterns of *L. welshimeri* and *L. booriae*.** Distance-decay relationship in *L. welshimeri* and *L. booriae* was inferred by the linear regression for genetic similarities measured by Jaccard similarity of gene absence/presence and geographic distances. A steeper negative slope with a higher *R^2^* indicates a stronger distance-decay relationship.

**Supplementary Figure 15. Comparison between soil and wild bird *L. welshimeri* isolates. (A)** Pairwise average nucleotide identity (ANI) comparisons between soil and wild bird isolates of *L. welshimeri*. **(B)** Genome size, gene richness, and GC content compared between monophyletic soil and wild bird *L. welshimeri* isolates. Box plots display the IQR with the median indicated as a line and whiskers extending to 1.5 times the IQR. Two-sided MW *U P* values are annotated.


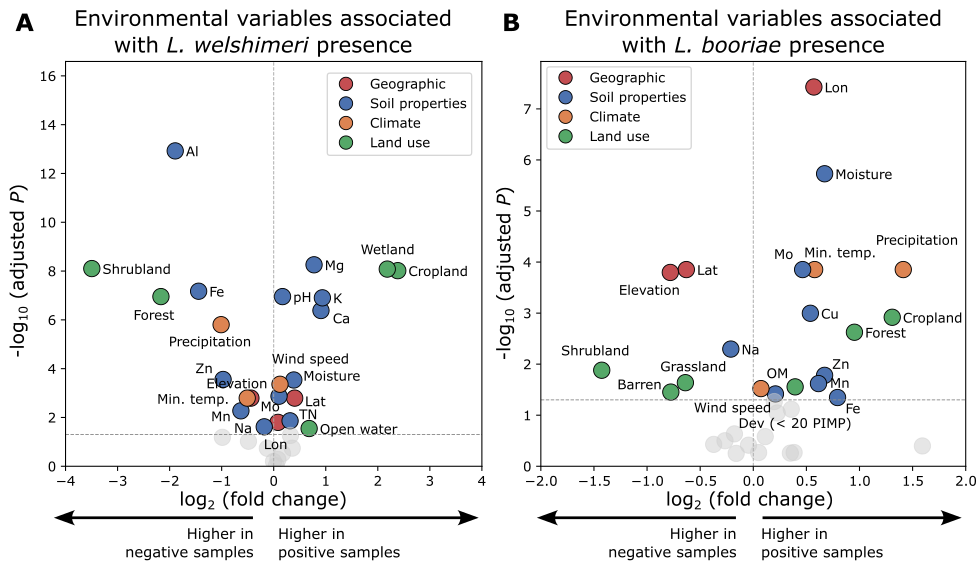


**Supplementary Figure 16. Comparison of abiotic environmental factors between sites positive and negative for *L. welshimeri* and *L. booriae***. **(A-B)** Volcano plots showing fold change in abiotic factors (x-axis) versus statistical significance (y-axis; adjusted two-sided MW *U P*) for **(A)** *L. welshimeri* and **(B)** *L. booriae*. Points above the gray dashed line indicate adjusted *P* < 0.05 and are color-coded by abiotic variable category, whereas non-significant abiotic factors are shown in gray**.** Abbreviations of abiotic factors are described in Methods.

**References**

1. Chen S. Ultrafast one‐pass FASTQ data preprocessing, quality control, and deduplication using fastp. *iMeta* 2023;**2**:e107. https://doi.org/10.1002/imt2.107

2. Bankevich A et al. SPAdes: A New Genome Assembly Algorithm and Its Applications to Single-Cell Sequencing. *J Comput Biol* 2012;**19**:455–477. https://doi.org/10.1089/cmb.2012.0021

3. Gurevich A et al. QUAST: quality assessment tool for genome assemblies. *Bioinformatics* 2013;**29**:1072–1075. https://doi.org/10.1093/bioinformatics/btt086

4. Bushnell B. BBMap: a fast, accurate, splice-aware aligner. *Lawrence Berkeley National Laboratory* 2014.

5. Li H et al. The Sequence Alignment/Map format and SAMtools. *Bioinformatics* 2009;**25**:2078–2079. https://doi.org/10.1093/bioinformatics/btp352

6. Chklovski A et al. CheckM2: a rapid, scalable and accurate tool for assessing microbial genome quality using machine learning. *Nat Methods* 2023;**20**:1203–1212. https://doi.org/10.1038/s41592-023-01940-w

7. Wood DE, Lu J, Langmead B. Improved metagenomic analysis with Kraken 2. *Genome Biol* 2019;**20**:257. https://doi.org/10.1186/s13059-019-1891-0

8. Hyatt D et al. Prodigal: prokaryotic gene recognition and translation initiation site identification. *BMC Bioinformatics* 2010;**11**:119. https://doi.org/10.1186/1471-2105-11-119

9. Steinegger M, Söding J. MMseqs2 enables sensitive protein sequence searching for the analysis of massive data sets. *Nat Biotechnol* 2017;**35**:1026–1028. https://doi.org/10.1038/nbt.3988

10. Murrell B et al. Gene-wide identification of episodic selection. *Mol Biol Evol* 2015;**32**:1365–1371. https://doi.org/10.1093/molbev/msv035

11. Martin DP et al. RDP4: detection and analysis of recombination patterns in virus genomes. *Virus Evol* 2015;**1**:vev003. https://doi.org/10.1093/ve/vev003

12. U.S. Geological Survey (USGS). Annual NLCD collection 1 science products: U.S. Geological Survey data release. 2024. https://doi.org/https://doi.org/10.5066/P94UXNTS

13. Bagi Z et al. Without borders? The impact of political barriers and land use on the animal health dynamics and genetic structures of large game species in the Carpathian Basin and surrounding regions—a systematic review. *Vet Sci* 2026;**13**:302. https://doi.org/10.3390/vetsci13030302

14. Zeller KA, McGarigal K, Whiteley AR. Estimating landscape resistance to movement: a review. *Landsc Ecol* 2012;**27**:777–797. https://doi.org/10.1007/s10980-012-9737-0

15. Wilkinson EB, Branch LC, Miller DL. Functional habitat connectivity for beach mice depends on perceived predation risk. *Landsc Ecol* 2013;**28**:547–558. https://doi.org/10.1007/s10980-013-9858-0

16. Fahrig L, Rytwinski T. Effects of roads on animal abundance: an empirical review and synthesis. *Ecol Soc* 2009;**14**:21. https://doi.org/10.5751/ES-02815-140121

17. Merrick MJ, Koprowski JL. Circuit theory to estimate natal dispersal routes and functional landscape connectivity for an endangered small mammal. *Landsc Ecol* 2017;**32**:1163–1179. https://doi.org/10.1007/s10980-017-0521-z

18. Aronson MFJ et al. A global analysis of the impacts of urbanization on bird and plant diversity reveals key anthropogenic drivers. *Proc R Soc B* 2014;**281**:20133330. https://doi.org/10.1098/rspb.2013.3330

19. Robb GN et al. Food for thought: supplementary feeding as a driver of ecological change in avian populations. *Front Ecol Environ* 2008;**6**:476–484. https://doi.org/10.1890/060152

20. Shochat E et al. From patterns to emerging processes in mechanistic urban ecology. *Trends Ecol Evol* 2006;**21**:186–191. https://doi.org/10.1016/j.tree.2005.11.019

21. Buler JJ, Moore FR. Migrant–habitat relationships during stopover along an ecological barrier: extrinsic constraints and conservation implications. *J Ornithol* 2011;**152**:101–112. https://doi.org/10.1007/s10336-010-0640-7

22. Guo F et al. Autumn stopover hotspots and multiscale habitat associations of migratory landbirds in the eastern United States. *Proc Natl Acad Sci USA* 2023;**120**:e2203511120. https://doi.org/10.1073/pnas.2203511120

23. Horton KG et al. Artificial light at night is a top predictor of bird migration stopover density. *Nat Commun* 2023;**14**:7446. https://doi.org/10.1038/s41467-023-43046-z

24. Loss SR et al. Bird–building collisions in the United States: estimates of annual mortality and species vulnerability. *Condor* 2014;**116**:8–23. https://doi.org/10.1650/CONDOR-13-090.1

25. Liao J et al. Nationwide genomic atlas of soil-dwelling *Listeria* reveals effects of selection and population ecology on pangenome evolution. *Nat Microbiol* 2021;**6**:1021–1030. https://doi.org/10.1038/s41564-021-00935-7

**Supplementary Tables for this manuscript include the following:**

**Supplementary Table 1.** Quality statistics of nine *L. welshimeri* wild bird isolates for whole-genome sequencing.

**Supplementary Table 2**. List of KEGG pathway identifiers used in pathway diversity analysis.

**Supplementary Table 3.** List of independent variables with variance inflation factors (VIF) below 10 used in the variation partitioning analysis (VPA), along with their corresponding model and variable categories.

**Supplementary Table 4.** Potential wildlife vectors for *Listeria* identified from published literature.

**Supplementary Table 5.** Predicted essential nutrient requirements inferred from genome-scale metabolic models.

**Supplementary Table 6.** Predicted utilizable substrates determined by flux balance analysis.

**Supplementary Table 7.** Functional classification of utilizable substrates showing significant differences between species.

**Supplementary Table 8.** List of genes under positive selection without evidence of homologous recombination.

**Supplementary Table 9.** Significant correlations between bacterial genera and gene richness, both overall and stratified by COGs.

**Supplementary Table 10.** Significant correlations between bacterial species and gene richness, both overall and stratified by COGs.

**Supplementary Table 11.** Linear regression analyses between genomic distance against landscape resistance across three spatial resolutions.
